# Endodontic length measurements using 3D Endo, cone-beam computed tomography, and electronic apex locator

**DOI:** 10.1186/s12903-021-01625-w

**Published:** 2021-05-18

**Authors:** Khoa Van Pham

**Affiliations:** Department of Operative Dentistry and Endodontics, Faculty of Odonto-Stomatology, University of Medicine and Pharmacy at Ho Chi Minh City, 652 Nguyen Trai Street, Ward 11, District 5, Ho Chi Minh City, Vietnam

**Keywords:** 3D Endo, Cone-beam computed tomography, Electronic apex locator, Endodontics, Root canal length

## Abstract

**Background:**

The objective of this study is to investigate the accuracy of the 3D Endo software, cone-beam computed tomography (CBCT) software, and the electronic apex locator (EAL) in endodontic length determination.

**Methods:**

302 root canals in 111 human extracted molars were chosen. Access cavity was performed, and root canal lengths were measured with a digital caliper for actual length (AL) and EAL for electronic length. Teeth were then scanned using CBCT device at voxel size of 0.10 mm. It measured root canal lengths using the CBCT (Romexis Viewer), 3D Endo for proposed length (3D-PL) and correct length (3D-CL). Mean differences between the four methods with the AL were calculated and compared. Fisher’s exact test, paired t-test, Bland-Altman plot were used to test the differences among the experimental modalities in working length determination at the significance of 0.05.

**Results:**

The accuracy in the range of ± 0.5 mm of the EAL ProPex II was highest among the experimental modalities, however this method disagreed with the actual length.

**Conclusions:**

The correct working length after adjustment from the semi-automatically length by the 3D Endo software and Romexis Viewer measurements agreed with the AL.

## Background

One of the most important phases in endodontic therapy is the root canal instrumentation, which is basically established on the working length (WL) determination [1]. An appropriate WL is utmost important in keeping the preparation inside restricted radicular space, prohibiting apical extrusion and securing good obturation [1]. The apical constriction is the ideal and practical point where the root canal procedure should be terminated, although this anatomic landmark does not exist in every case [2].

Periapical (PA) radiograph has been the most conventional modality for reliable and standard WL determination in many dental schools in the world. However, this analogue or digital film has certain shortcomings, leading to misinterpret the actual situation, identify root apex incorrectly, distort image, and overestimate WL [1].

After the unsuccess of early few generations of electronic apex locator (EAL), the major shortcomings of these EALs are overcome by the contemporary EALs with the coming out of “multiple frequencies” or “ratio method” [3]. Although the EAL overcomes the radiograph in reliability and accuracy, its performance might be falsified by several circumstances such as lack of patency, electric conductivity of restoration, or complexities in anatomical configuration [4].

There is no individual modality that completely satisfies all requirements of working length determination.

Cone-beam computed tomographic (CBCT) image is a contemporary radiographic imaging system and overcomes several shortcomings encountered with traditional radiographic modalities [5]. Information obtained from the preexisting CBCT scan allows for proper diagnosis, appropriate treatment planning, and predictable canal management [5]. Geometrically precise measurement tools are helpful in establishing intra- and inter-canal distance relationships and determination of root canal length [6]. Reports from several studies in the literature regarding the precision of CBCT measurements compared with that of conventional radiographs or EALs reach no concurrence [1, 7, 8].

Recently, 3D Endo software (Dentsply Sirona, Johnson City, TN, USA) has been introduced for complex cases of endodontic treatment planning [9]. The software uses input CBCT data of standard DICOM with minimum resolution of 200 μm and offers an intuitive, lively, attractive interface for analysis. An innovative and creative feature of the 3D Endo software is the capability of creation of the pathway of the canal semi-automatically, after the orifice and apical foramen is defined by the operator. Based on this pathway of the canal, a virtual file is automatically inserted into the canal and suggested WL is offered by the software. In the case of unsatisfied suggested length, the virtual rubber stop can be adjusted to the more appropriate reference landmark on the occlusal or incisal of the tooth by the operator.

The objective of this study is to investigate the accuracy of the 3D Endo software, CBCT software (Romexis Viewer, Planmeca Oy, Helsinki, Finland), and the EAL ProPex II (Dentsply Sirona, Ballaigues, Switzerland) in WL determination.

## Materials and methods

The present study was approved by the Research Ethics Committee of the University of Medicine and Pharmacy at Ho Chi Minh City, Vietnam. The approval number of the study was 3707/QĐ-SĐH. The study acquired the intact human extracted molars obtained from many hospitals for many reasons. Using the data from the previous study [1], and the sample size calculation in Bland-Altman plot submenu of the MedCalc Statistical Software version 19 (MedCalc Software, Ostend, Belgium), the size was 302 root canals. Therefore, 111 extracted molars were chosen for the present study. Teeth were cleaned using the ultrasonic scaler BobCat (Dentsply Sirona, Switzerland), immersed in the 10% formalin solution. Teeth were observed thoroughly under a stereomicroscope at a magnification of ten to exclude immature apical, cracked, external resorption roots.

The teeth were cleaned with saline and prepared for accessing. After being coded with numbers on the crowns, the access cavity was prepared with the straight-line access concept using the Martin and Endo-Z burs (Dentsply Sirona, Ballaigues, Switzerland). After exposure of all canal orifices was completed, the #10 ISO K-file was introduced into all canals until the tip of the file was visible at the most coronal border of the AF opening under the stereomicroscope (Olympus SZX16, Olympus Corp., Tokyo, Japan). The rubber stop was adjusted to the occlusal reference point, the file was removed from the canal and the length from tip to rubber stop of the file was measured using a digital caliper Mitutoyo (Mitutoyo Corp, Kawasaki, Japan) and recorded as the actual canal length (AL).

The teeth were immersed in the freshly mixed alginate tray to prepare for electronic measurements. The root canal lengths were measured using the electronic apex locator ProPex II (Dentsply Sirona, Ballaigues, Switzerland). The #10 ISO K-file was inserted into the canal until the 0.0 mark lighted up and remained stable for 5 s. The rubber stop was adjusted to the occlusal reference point, the file was withdrawn from the canal and the length was measured as mentioned above for AL. This length was recorded as the electronic length (EL).

The teeth were arranged and immersed into plastic mold containing 3 mm thick floor wax and remained light impression silicone on top that reached to the cemento-enamel junctions. Molds containing teeth were then scanned using the cone beam computed tomography (CBCT) (Planmeca ProMax, Planmeca Oy, Helsinki, Finland) with endo mode, 90 kV, 10 mA, field of view 50 × 50 mm, voxel size of 0.10 mm.

**Fig. 1:**
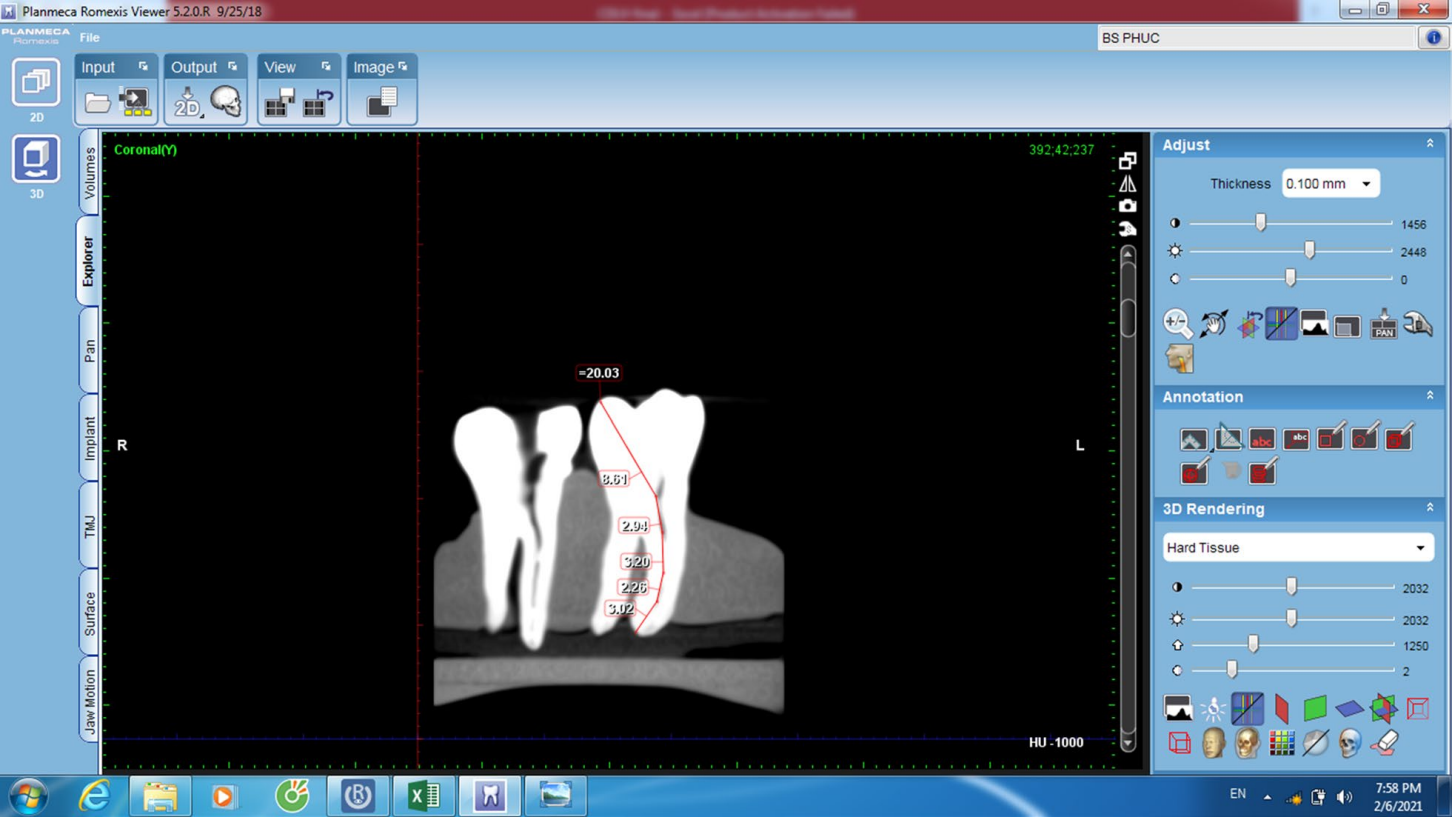
Romexis Viewer measurement on the CBCT software

The CBCT images were scanned and analyzed using the Romexis Viewer software from CBCT manufacturer with the slice thickness of 1 mm and the interval of 0.1 mm. The slices of the tooth were scanned and analyzed in both of bucco-lingual and mesio-distal views to make sure there was no canal curvature missed from measurements. The slice with the best image of entire length of the canal in bucco-lingual view at the greatest curved angle was selected. The measuring line was drawn from the occlusal reference to the apical foramen (AF), accompanying any deviations from the course of the canal, and was measured in millimeters. Root canal lengths were measured using the tools of Romexis Viewer and recorded as the CBCT length (Fig. 1). The CBCT measurements were performed twice with an interval of two weeks to check the intra-examiner reliability.

**Fig. 2:**
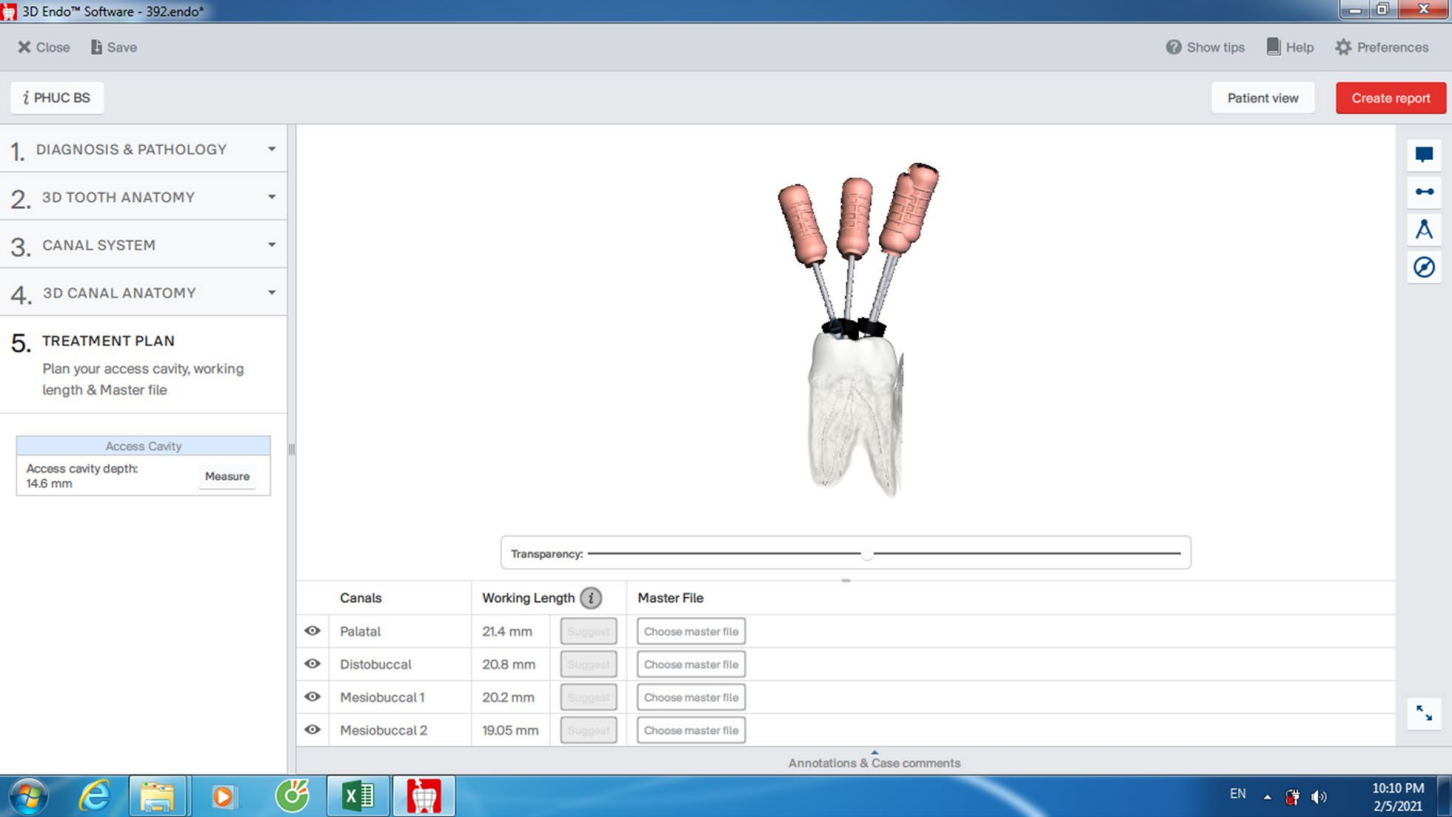
Proposed length measurement on the 3D Endo software

The CBCT data then were opened on the 3D Endo software (Dentsply Sirona, Salzburg, Austria) to analyze using its own tools. Tooth’s 3-D image was isolated with surrounding material using the tools of the software. Once the canal orifice and the apex foramen were defined for each canal of each tooth, the automatic line was drawn by software to connect these two landmarks. The pathway of the canal was defined by selecting and adjusting the positions of as many points as possible on this line in horizontal and vertical planes from the orifice to apex. The 3-D image of tooth and canal system inside was automatically reconstructed and K-files were automatically inserted into the canals reaching to the apices. After adjustment of the coronal angulation of the file following the straight-line access concept, the proposed length (3D-PL) was automatically created by pressing the Suggest button on the interface of software and was recorded as 3D-PL (Fig. 2). Normally, the position of the rubber stop on the occlusal surface at the proposed length (PL) was not appropriate to the operator, therefore, adjustment of the rubber stop position was conducted by operator for best suitable location and this length was recorded as correct length (3D-CL). The 3D Endo measurements were performed twice with an interval of two weeks to check the intra-examiner reliability.

All Romexis Viewer and 3D Endo CBCT assessment and mensuration were realized by the same endodontist especially trained in CBCT image and 3D Endo application using the Dell Latitude E7440 system (Dell Technologies, USA).

All measuring data were stored and analyzed using MedCalc Statistical Software version 19 (MedCalc Software, Ostend, Belgium). The data were first screened for normality of distribution using the Shapiro-Wilk test. The intra-examiner reliability was checked using IntraClass Correlation (ICC) index. Fisher’s exact test, Paired t-test, ICC indices and Bland-Altman plots were used for analyzing the data.

## Results

The intra-examiner ICC indices were greater than 0.96 for all relevant measurements for the present study.

The proportions (%) of differences between the four experimental modalities and the AL measurements were the Table 1. The proportions of differences when using the 3D Endo PL, CL, Romexis Viewer, and ProPex II were 86.4%, 92.8%, 64.3%, and 100%, respectively.

**Table 1:** The incidence (%) of differences between the four methods and the AL measurements

Groups	Shorter than AL N(%)		Equal to AL N(%)	Longer than AL N(%)		± 0.5 mm(%)
	> 0.5 mm	≤ 0.5 mm		≤ 0.5 mm	> 0.5 mm	
3D-PL–AL	13 (4.3)	85 (28.1)	19 (6.3)	157 (52.0)	28 (9.3)	86.4^a^
3D-CL–AL	14 (4.6)	133 (44.0)	14 (4.6)	133 (44.0)	8 (2.6)	92.8^b^
Romexis Viewer–AL	56 (18.5)	73 (24.2)	0 (0.0)	121 (40.1)	52 (17.2)	64.3^c^
ProPex II–AL	0 (0.0)	299 (99.0)	3 (1.0)	0 (0.0)	0 (0.0)	100^d^

^a,b,c,d^Different superscript letters showed that there were significant differences at the level of 0.05 (P < 0.05), Fisher’s Exact Test

**Fig. 3:**
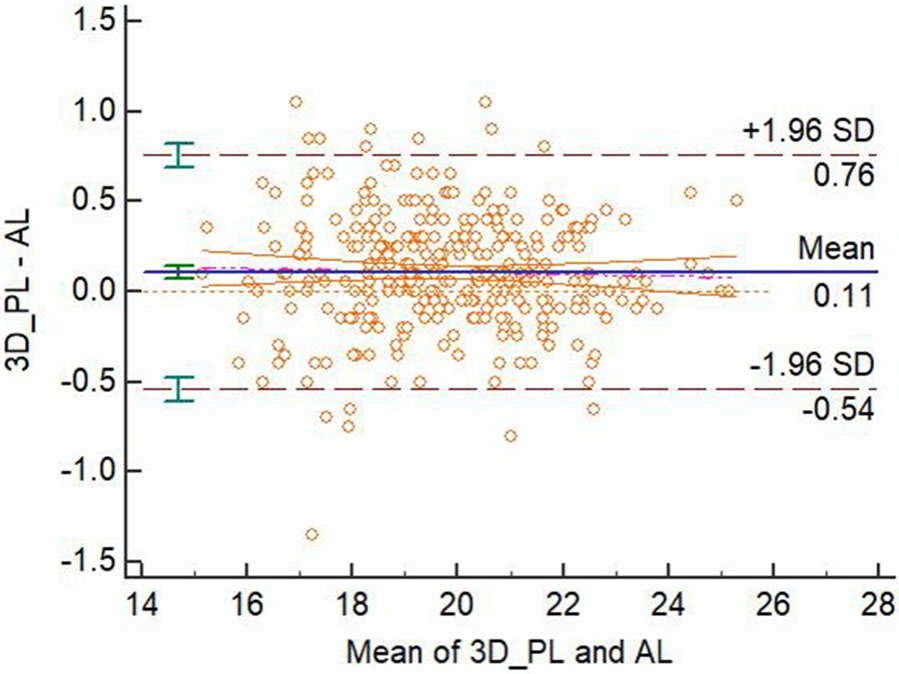
Bland-Altman plot for the agreement of 3D-PL and AL measurements

**Fig. 4:**
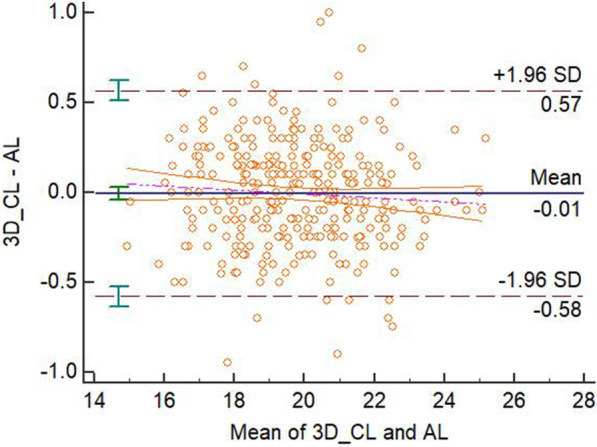
Bland-Altman plot for the agreement of 3D-CL and AL measurements

**Fig. 5:**
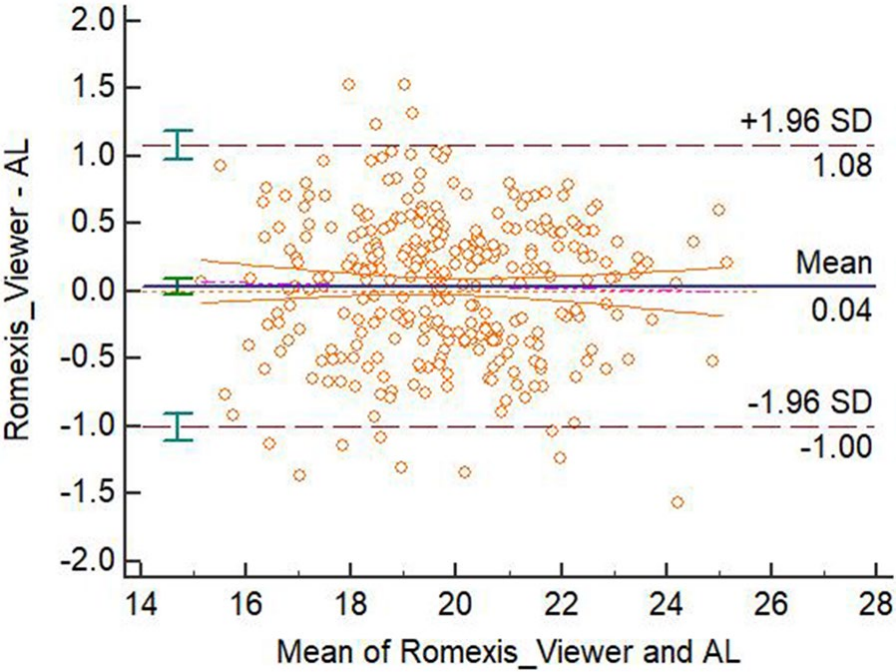
Bland-Altman plot for the agreement of Romexis Viewer and AL measurements

**Fig. 6:**
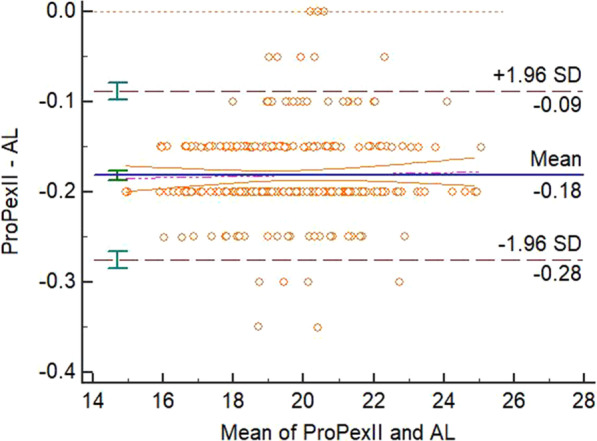
Bland-Altman plot for the agreement of ProPex II and AL measurements

The mean biases, confidence intervals, *P* values in the paired t-test and linear regression analysis, fixed or proportional biases for different methods’ measurements were displayed in the Table 2. There were significant differences between the 3D-PL or ProPex II and the AL measurements in the paired t-test and there was not significant difference in the linear regression analysis of these two modalities. With the analysis from the previous studies [10, 11], this means that there were fixed biases between paired measurements without proportional bias, therefore these two modalities disagreed with the AL. There was not a significant difference in both paired t-test and linear regression between the 3D-CL or Romexis Viewer and the AL measurements. Therefore, these two modalities agreed with the AL. The Bland-Altman plots were displayed in the four figures, from Figs. 3, 4, 5 and 6.

**Table 2:** Mean biases, confidence intervals, *P* values in two statistical tests, fixed or proportional biases for different methods’ measurements

Groups	Paired t-test			Linear regression	Fixed bias	Proportional bias
	Mean bias	95% CI	*P*	*P*		
3D-PL–AL	0.1076	0.0701 to 0.1451	< 0.0001*	0.6565	Yes	No
3D-CL–AL	− 0.0056	− 0.0386 to 0.0274	0.7375	0.2291	No	No
Romexis Viewer–AL	0.0383	− 0.0218 to 0.0986	0.2112	0.6357	No	No
ProPex II–AL	− 0.1815	− 0.1869 to − 0.1761	< 0.0001*	0.5733	Yes	No

*Differences at a significant level of 0.0001, Paired t-test

## Discussion

The results showed that the ProPex II was the best measurements with the highest proportion of accuracy in the range of ± 0.5 mm. However, this EAL disagreed with the AL. There were two modalities agreed with the AL, using the CBCT data. Both of them could be considered for alternators for root canal determination.

3D Endo software is developed, especially dedicated to endodontic therapy in the clinical setting. However, with the friendly, intuitive interface and thoroughly clear instructions step-by-step throughout procedure, 3D Endo completely satisfies all requirements from simple to complex cases, especially in the pre-clinical endodontic education. WL determination is one of the most innovative features of the 3D Endo with the function of manual adjusted length by operator should the suggested length be not satisfied. This feature is developed in the effort of maximum reduction of operator’s errors in WL determination. Depending on the curvature levels of the canal after adjusting the pathway of the canal using the appropriate slices in the 3D Endo, the operator can estimate proper length of instrumented canal to correct the WL at ultimate steps. The virtual pathway of the canal lively intuitively displayed on the screen of the computer assists the operator in effective visualization and management of the root canal instrumentation. Although there were many advantages in using the 3D Endo software, the result showed that the proposed length of the program disagreed with the AL. With the voxel size of 0.10 mm, the resolution of the image acquired from the CBCT device might appropriate for WL determination, however, accessed teeth were used for evaluation might lead to occlusal structure missing on the reconstructed image and might lead to inaccurate determination by the software. The result showed that the correct length by the operator agreed with the AL and obtained the highest accuracy between ± 0.5 mm in three CBCT data modalities.

The human extracted teeth are commonly used for studies using CBCT in WL determination in dry mandible or in jaw model [12–14]. The setting with the dry mandible is better than other design in controlling of certain clinical variables such as artifacts caused from position or motion of patient, beam hardening from other materials, or noise from other anatomic structures [12, 14]. The arrangement of teeth in the impression tray of the present study induces certain artifacts from the neighboring teeth in the tray. However, CBCT images are clear and anatomic landmarks are defined easily and exactly with no interferences. Although the human extracted teeth seem appropriately for evaluation the accuracy of CBCT WL determination, the artificial endodontic training tooth still completely satisfies requirements of this purpose [15]. Authors of that study just select the actual root canal length of the artificial tooth as the gold standard in evaluation the accuracy of the CBCT WL without EAL measurements [15]. The 3D Endo software can enhance accurate 3D root canal length determination, however, the working length has to be checked, controlled, and maintained continuously during the preparation phase to detect possible length changes, especially in the severe curved canal [15].

CBCT is an added method for determination of WL, particularly valuable with retreatment when removing gutta-percha to save time and prevent over-instrumentation [6]. One of the important shortcomings when using the CBCT for endodontic WL determination on the heavily metallic restored tooth is the significant artifact [6]. More artifact means a greater approximate range of length, and in these cases, CBCT provides only an estimate of the length. Sometimes neighboring structure assists in estimation of an approximate length [6]. In the present study, the Romexis Viewer measurements agreed with the AL, although the accuracy in the range of ± 0.5 mm was lowest among all other methods. The voxel size of 0.10 mm in the present study seemed appropriate for endodontic length measurements with the acceptable result.

With the advance of technology in production of more and more modern root canal instruments [16, 17], and the complexity of the root canal morphology [18], the more important role of the endodontic length determination is.

Knowledge of root canal anatomy and morphology is essential for every clinician in endodontics in identifying the root canal orifices. CBCT imaging has offered a precise, noninvasive, real-time approach for clinical chairside evaluation of root canal anatomy and morphology [5]. The 3D Endo software, dedicated endodontic program using CBCT data, is an effective, quick, and easy modality for identification and visualization of canal trajectories and confluences in three dimensions. This Endo software shows promise in help for operators quantifying anatomical complexities preoperatively [19].

Diagnostic examinations should be performed at the lowest dose of radiation, following the ALARA principle: as low as reasonably achievable [20]. Therefore, CBCT scans should only be performed when indicated, and consideration should be given to alternative modalities. The American Association of Endodontists statement suggests that the risk-benefit ratio is too high for CBCT to be used as a screening tool, even though the radiation levels are low with focused-field imagers [21]. Therefore, CBCT scans used as a routine procedure for endodontic diagnostic should be strictly indicated and application of CBCT only for root canal length measurement is not recommended [15]. In cases with the pre-existing CBCT owing to the other reasons but endodontics, the 3D Endo software is an invaluable instrument in root canal morphological investigation, treatment planning, and especially in working length determination [15].

The EAL still had the highest accuracy in the range of ± 0.5 mm, as shown by the result of the present study. However, this modality disagreed with the AL that could lead to overemphasize this device’s capability.

## Conclusions

The accuracy in the range of ± 0.5 mm of the EAL ProPex II was highest among the experimental modalities. The correct working length after adjustment from the semi-automatically length by the 3D Endo software and Romexis Viewer measurements agreed with the AL.

## Data Availability

The datasets used and/or analyzed during the current study are available from the corresponding author on reasonable request.

## Abbreviations

3-D: Three dimensions
AL: Actual length
CBCT: Cone beam computed tomography
CL: Corrected length
EAL: Electronic apex locator
EL: Electronic length
ICC: IntraClass correlation
PL: Purposed length
WL: Working length
